# Classification of Unifloral Honeys from SARDINIA (Italy) by ATR-FTIR Spectroscopy and Random Forest

**DOI:** 10.3390/molecules26010088

**Published:** 2020-12-27

**Authors:** Marco Ciulu, Elisa Oertel, Rosanna Serra, Roberta Farre, Nadia Spano, Marco Caredda, Luca Malfatti, Gavino Sanna

**Affiliations:** 1 Department of Animal Sciences, University of Göttingen, Kellnerweg 6, 37077 Göttingen, Germany; elisa.oertel@uni-goettingen.de; 2 Dipartimento di Chimica e Farmacia, Università degli studi di Sassari, Via Vienna 2, 07100 Sassari, Italy; rosanna1981@live.it (R.S.); roberta.farre@tiscali.it (R.F.); nspano@uniss.it (N.S.); lucamalfatti@uniss.it (L.M.); sanna@uniss.it (G.S.); 3 AGRIS Sardegna, Loc. Bonassai S.S. 291 Km 18.6, 07100 Sassari, Italy; caredda.m@gmail.com

**Keywords:** honey discrimination, strawberry-tree, thistle, eucalyptus, asphodel, attenuated total reflectance, Fourier transform infrared spectroscopy

## Abstract

Nowadays, the mislabeling of honey floral origin is a very common fraudulent practice. The scientific community is intensifying its efforts to provide the bodies responsible for controlling the authenticity of honey with fast and reliable analytical protocols. In this study, the classification of various monofloral honeys from Sardinia, Italy, was attempted by means of ATR-FTIR spectroscopy and random forest. Four different floral origins were considered: strawberry-tree (*Arbutus Unedo* L.), asphodel (*Asphodelus microcarpus*), thistle (*Galactites tormentosa*), and eucalyptus (*Eucalyptus calmadulensis*). Training a random forest on the infrared spectra allowed achieving an average accuracy of 87% in a cross-validation setting. The identification of the significant wavenumbers revealed the important role played by the region 1540–1175 cm^−1^ and, to a lesser extent, the region 1700–1600 cm^−1^. The contribution of the phenolic fraction was identified as the main responsible for this observation.

## 1. Introduction

Since prehistoric times, honey has been one of the most popular foods for humans. In addition to its role as a food sweetener, several studies have classified honey as functional food because of its several biological and nutraceutical properties such as antioxidant, anti-ulcer, antibacterial and also anti-tumor activities [1]. The distinct organoleptic properties combined with its nutritional characteristics constitute the basis of a continuously growing demand for honey. In recent years, honey imports into the EU have increased at a rate of more than 10,000 tons per year [2]. The occurrence of fraudulent activities aimed at placing on the global market honey mislabeled with regard to its floral origin or adulterated with exogenous sugars have prompted the European Union to implement control measures. However, the official analytical procedures provided by current legislation present several limitations in the identification of the botanical and/or geographical origin of honey [3]. In addition, the traditional melissopalinological analysis is not effective in the authentication of filtered honeys and those whose pollen is underrepresented. For these reasons, for several years the scientific community has been developing instrumental analytical protocols aimed at relating specific markers or classes of compounds to the floral origin of honey, most of them summarized in a number of authoritative reviews [4,5,6]. In this context, chromatographic techniques have for years been a key tool in the search for specific markers. For instance, the use of HPLC-based protocols has in the past made possible to identify the origin of honey through the profile of carbohydrates [7,8,9], phenolic compounds [7,10,11], and macro- and micro-nutrients [12]. In addition, the use of gas-chromatography (especially when coupled to mass spectrometry) has proved to be fundamental for the identification of volatile compounds related to honey source [13,14,15,16]. As regards the use of the volatile fraction as a tool for botanical origin attribution, non-chromatographic techniques have been also used, such as MS-based electronic nose [17]. 

Some studies have demonstrated the effectiveness of spectroscopic approaches directed rather than the quantification of specific compounds, to the acquisition of fingerprints containing the information necessary for the identification of honeys. Among these, Raman spectroscopy [18,19], NMR [20,21], VIS/NIR spectroscopy [22], and mass spectrometry [23] are worthy of citation. 

Attenuated total reflectance-Fourier transform infrared spectroscopy (ATR-FTIR) has proven to be particularly useful for the detection of honey adulteration [24,25,26,27,28]. Besides, scientific literature offers various examples related to the employment of this technique in the classification of botanical origin of honey. For instance, the differentiation of honeys of various floral origins from India [29], Turkey [30], Poland [31], and Croatia [32] was performed by means of ATR-FTIR combined with chemometrics.

Strawberry-tree honey (*Arbutus unedo* L.) represents one of the most typical beekeeping products of Sardinia, Italy, and, more generally, of the Mediterranean region [33,34]. The refined bitter taste along with the scarce production make this honey one of the most expensive of Southern Europe [35]. In addition, the presence in high amounts of various bioactive compounds (e.g., phenolic acids, flavonoids, terpenes etc.) gives strawberry-tree honey distinct nutraceutical and functional properties [36,37] comparable in some cases to those of the more famous Manuka honey [34]. The authentication of this honey has so far mainly been based on the qualitative and quantitative determination of its chemical marker, the homogentisic acid [10,38,39,40]. Other typical honeys of the area include asphodel (*Asphodelus microcarpus*), thistle (*Galactites tormentosa*), and eucalyptus (*Eucalyptus calmadulensis*) honeys [41,42]. Also for these products, the authentication of the botanical origin has been until now performed by the application of instrumental protocols aimed at identifying and/or quantifying specific chemical markers [33,43,44]. 

Multivariate data analysis and machine learning techniques have proved to be excellent strategies for honey discrimination [45]. Random forest is a classification algorithm based on the construction on several decision trees. A subset of independent features is used for the training phase in order to construct each tree. The deriving set of trees is then used to assign one class to an object (sample) on the basis of the most frequent classification among them [22,45,46,47]. The prediction accuracy obtained by a multitude of decision tree is generally higher than the one obtained with a single tree [46]. Despite the potential of this classification algorithm, only a few examples of the application of random forest for honey classification can be found in the literature [22,46,48]. 

To the best of our knowledge, a classification of the strawberry-tree honey using FTIR methods has been only once attempted but the low number of samples analyzed prevented from any reliable conclusion [49]. In this work, we report a classification of strawberry-tree honey along with three other typical floral origins from Sardinia (Italy) by means of ATR-FTIR spectroscopy combined with random forest. 

## 2. Results

### 2.1. ATR-FTIR Spectra

**Figure 1:**
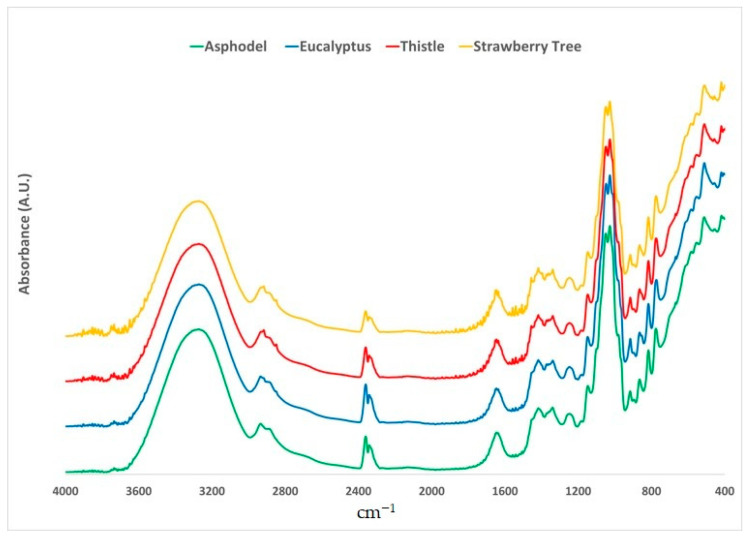
Average raw ATR-FTIR spectra of the selected floral origins.

Figure 1 shows raw representative ATR-FTIR spectra of the four selected honey types in the region 4000–400 cm^−1^. 

The visual analysis of the spectra allowed identifying the characteristic absorption bands of honey, based on the information provided by scientific literature [31]. More specifically, the region between 3000 and 2800 cm^−1^ includes the signals deriving from C-H stretching of carbohydrates [50], O-H stretching of carboxylic acids [51] and NH_3_ stretching of free amino acids [50,52]. Bands in the region 1700–1600 cm^−1^ are instead attributable to the O-H stretching and bending of water [53], the stretching of carbonyls mainly belonging to carbohydrates [50] and the N-H bending of primary amides of proteins [54]. In the spectral region 1540–1175 cm^−1^ it is possible to observe the absorption bands related to the stretching and bending of not water-related hydroxyl groups [50,55], C-O and C-H stretching of carbohydrates [56], and the carbonyl stretching of ketones [55]. Ring vibrations (mainly attributable to carbohydrates) [50,55] along with the signals related to C-O and C-C stretching are visible in the region between 1175 and 950 cm^−1^ [56,57]. Finally, between 940 and 700 cm^−1^, there is the anomeric region of carbohydrates [57,58] where the C-H bending [50,55,59] and ring vibrations produce signals [55]. 

### 2.2. Random Forest Classification

Across 100 runs, the random forest achieved a mean accuracy of 87% with a standard variation of 7%. The analysis was repeated with permuted labels. In this case, mean accuracy only reached 43%, indicating the former average accuracy was due to a real signal and no statistical artifact. Specificity and sensitivity values were 94.3% and 72.6% for asphodel, 93.9% and 87.3% for eucalyptus, 96.4% and 90.5% for thistle, and 99.9% and 91.6% for strawberry-tree, respectively. 

For each run, the ten most important wavenumbers were identified. Table 1 lists preselected ranges and the frequency at which the most important wavelengths fell into them.

## 3. Discussion

One of the greatest advantages of the adopted approach is given by the total absence of any sample pre-treatment along with the possibility to obtain, for each one of the samples, all the information required to build the classification model in a few seconds. To the best of our knowledge, this study provides the first example of the building of a machine learning model aimed at predicting the selected honey botanical sources without the support of any sample extraction and/or clean-up. The combination of ATR-FTIR with random forest algorithm proved to be successful for the classification of the selected floral origins. Although 87% prediction accuracy could be considered, in some data science scenarios, relatively low when k-fold or leave-one-out cross validation is performed, it is important to bear in mind that the above mentioned cross validation approaches tend to overestimate the general model performances when applied on small datasets [60]. The lack of previous studies aimed at classifying the selected floral origins by means of IR spectroscopy and random forest prevents us to make reasonable comparisons regarding the model accuracy. As regards the application of random forest in combination with other analytical techniques for honey classification, to the best of our knowledge only two contributions have been until now published. In the first study, electrophoresis was used in order to discriminate between two different honey types [48]. In the most recent contribution, the classification of honeys belonging to the different floral sources was successfully achieved (prediction accuracy of 98.2%) by means of laser induced breakdown spectroscopy and random forest [61]. However, in this study only ten samples were considered, and the specific attribution of the various floral sources was somehow missing, being some of them simply indicated as “flower honey” or “forest honey”. 

**Figure 2:**
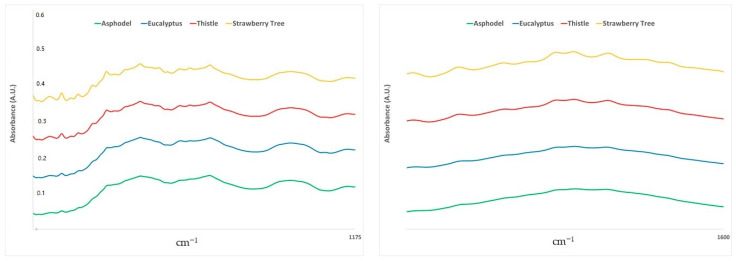
Average ATR-FTIR spectra in the 1540–1175 cm^−1^ (left) and 1700–1600 cm^−1^ (right) regions.

Our results further support the importance of the so-called fingerprint region in the definition of the floral origin of honey. In fact, also in previous studies [22,30], the interval 1800–750 cm^−1^ played a predominant role in the differentiation of honeys, although in those cases no classification technique was applied (i.e., only principal component and/or hierarchical cluster analysis). In our case, the major contribution of the various wavenumbers can be traced back to an even narrower range (1540–1175 cm^−1^, 87%). As already explained above, the absorptions in this spectral region are mainly due to the stretching and bending of not water-related hydroxyl groups, C-O and C-H stretching of carbohydrates and the carbonyl stretching of ketones [30]. Flavanols and phenols contribute to this spectral region [62]. This observation is somehow supported by the conclusions obtained in a previous study, where total content of polyphenols was considered, among various chemical and physical parameters, one of the main discriminant factors between strawberry-tree, asphodel, thistle and eucalyptus honeys from Sardinia [42]. Although to a much lesser extent, also the 1700–1600 cm^−1^ range contributed to the classification of the four types of honey (10.7% of the wavenumbers). The presence of phenolic compounds has been in the past related to the bands in this region, supporting the hypothesis that the profile of polyphenols could underlie this honey differentiation. Average spectra in these regions are shown in Figure 2. 

**Figure 3:**
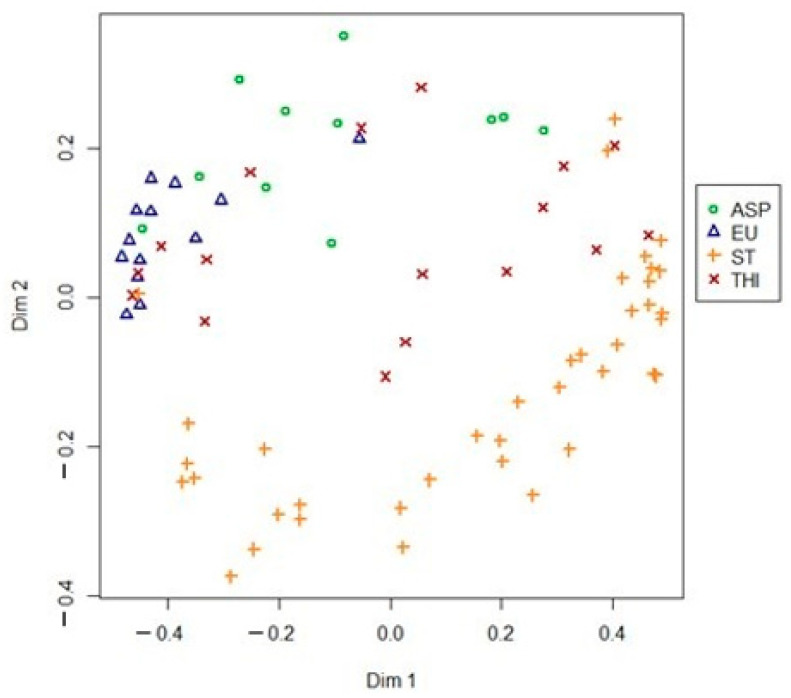
2D visualization of unsupervised random forest.

As a mere visualization aid, an unsupervised (i.e., with no classification and/or regression aims) random forest was performed and plotted after multidimensional scaling (MDS, Figure 3). 

The unsupervised proximity information plotted in 2D shows how strawberry-tree honeys are clearly identifiable, being the corresponding cluster visibly distinguishable from the other three. This observation could be the evidence of the influence of the distinct chemical characteristics of strawberry-tree honey on the IR spectrum. In fact, this honey stands out from the other local honeys due to its extremely high polyphenol content, which constitutes the main reason for its nutraceutical properties. This observation is also supported by the high sensitivity value recorded for strawberry-tree honeys, very close to 100%. On the other hand, asphodel honeys showed the lowest sensitivity resulting as the most misclassified among the four botanical origins, over the 100 iterations. These findings could be explained by the wide variability in the chemical composition of asphodel honey [33]. For example, in a previous study concerning the classification of the same floral sources by physicochemical determinations, this honey showed, compared to the others, a wider range in the total phenolic content, FRAP antioxidant activity and the DPPH radical scavenging activity [42]. Also, eucalyptus honeys showed a sensitivity lower than 90%. However, this case should be otherwise considered in comparison to the one just mentioned, since this lower sensitivity value can be attributable to one single eucalyptus honey sample which was repeatedly misclassified as asphodel honey over several cross-validation iterations, revealing a likely initial mislabeling.

## 4. Materials and Methods

### 4.1. Honey Samples

A total of 80 honey samples was collected from beekeepers from Sardinia (Italy) in the corresponding harvesting season (early spring for asphodel honey, late spring for thistle honey, summer for eucalyptus honey and autumn for strawberry tree honey). All samples were stored in the dark at 4 °C until analysis. The assignment of floral origin was accomplished on the basis of the information provided by local producers and melissopalinological analysis that provided, for each sample, data within the range measured by Floris et al. [41].

### 4.2. ATR-FTIR Spectroscopy

ATR-FTIR spectra were acquired by means of a Vertex 70 spectrophotometer equipped with a platinum ATR-QL diamond accessory (Bruker Optics, Ettlingen, Germany). Spectra were acquired in the region 4000–400 cm^−1^ by averaging 256 scans with a resolution of 4 cm^−1^, including background subtraction of the diamond window. The diamond was cleaned between samples by using ethanol and ultrapure water.

### 4.3. Random Forest

A random forest was used in a cross-validation setting, where 70% (n = 56) of the samples were randomly chosen as training set, the remaining 30% (n = 24) were used for internal validation. More specifically, a shuffle-split cross-validation over 100 iterations was performed. The typical honey wavenumber intervals already described in Section 2.1 as features were used. Thus, from the original 3800 features/wavelength, a subset of 1182 remained. Selection of the most significant features/wavenumbers was achieved based on the highest mean decrease in accuracy per cross validation iteration. All statistical analysis was conducted in R with the package “randomForest” [63].

## 5. Conclusions

Given the paucity of contributions aimed to evaluate the potential of the combination of ATR-FTIR spectroscopy and random forest to classify honeys, this approach has been used on a representative sampling of the most renowned four unifloral honeys from Sardinia, Italy. The results demonstrated for a good level of prediction accuracy, obtaining for each sample the required analytical information in a short time. Aspects like a difficult classification on a palynological basis and the wide variability in the chemical composition of the asphodel honey should be taken into account when this botanical origin is included in the classification model. On the basis of the results reported here, further studies are required to assess the prediction accuracy of this approach for a larger number of botanical origins. As a final remark, since the phenolic fraction has been a key parameter for discriminating the selected honeys on the basis of the infrared spectra, attention will be paid in the future to the contribution of polyphenols towards the IR absorption spectra of unifloral honeys.

## Figures and Tables

**Table 1:** Distribution of important wavelengths across preselected ranges.

Wavenumber Range (cm^−1^)	Frequency (%)
3000–2800	1.2
1700–1600	10.7
1600–1540	1.1
1540–1175	87
1175–940	0
940–700	0

## Data Availability

The data used to support the findings of this study are available from the corresponding author upon request.

## Sample Availability

Honey samples used for this study are not available from the authors.
